# Mesenchymal Stromal Cell-Derived Extracellular Vesicles – Silver Linings for Cartilage Regeneration?

**DOI:** 10.3389/fcell.2020.593386

**Published:** 2020-12-10

**Authors:** Andrea De Luna, Alexander Otahal, Stefan Nehrer

**Affiliations:** Center for Regenerative Medicine, Department for Health Sciences, Medicine and Research, Danube University Krems, Krems an der Donau, Austria

**Keywords:** mesenchymal stem cell, extracellular vesicle, osteoarthritis, biomarkers, *in vitro* studies and *in vivo* studies

## Abstract

As the world’s population is aging, the incidence of the degenerative disease Osteoarthritis (OA) is increasing. Current treatment options of OA focus on the alleviation of the symptoms including pain and inflammation rather than on restoration of the articular cartilage. Cell-based therapies including the application of mesenchymal stromal cells (MSCs) have been a promising tool for cartilage regeneration approaches. Due to their immunomodulatory properties, their differentiation potential into cells of the mesodermal lineage as well as the plurality of sources from which they can be isolated, MSCs have been applied in a vast number of studies focusing on the establishment of new treatment options for Osteoarthritis. Despite promising outcomes *in vitro* and *in vivo*, applications of MSCs are connected with teratoma formation, limited lifespan of differentiated cells as well as rejection of the cells after transplantation, highlighting the need for new cell free approaches harboring the beneficial properties of MSCs. It has been demonstrated that the regenerative potential of MSCs is mediated by the release of paracrine factors rather than by differentiation into cells of the desired tissue. Besides soluble factors, extracellular vesicles are the major component of a cell’s secretome. They represent novel mechanisms by which (pathogenic) signals can be communicated between cell types as they deliver bioactive molecules (nucleic acids, proteins, lipids) from the cell of origin to the target cell leading to specific biological processes upon uptake. This review will give an overview about extracellular vesicles including general characteristics, isolation methods and characterization approaches. Furthermore, the role of MSC-derived extracellular vesicles in *in vitro* and *in vivo* studies for cartilage regeneration will be summarized with special focus on transported miRNA which either favored the progression of OA or protected the cartilage from degradation. In addition, studies will be reviewed investigating the impact of MSC-derived extracellular vesicles on inflammatory arthritis. As extracellular vesicles are present in all body fluids, their application as potential biomarkers for OA will also be discussed in this review. Finally, studies exploring the combination of MSC-derived extracellular vesicles with biomaterials for tissue engineering approaches are summarized.

## Introduction

As Osteoarthritis (OA) is one of the fastest growing major health condition especially in the aging population, development of new therapeutic approaches for osteochondral regeneration is needed. Till now, treatment options for OA are mainly palliative focusing on relieve of pain and inflammation, but do not result in a fully restoration of the joint’s mechanical function. In advanced stages of OA, total arthroplasty is the only option but is also linked to drawbacks including donor morbidity and limited implant lifetime. Besides using unaffected chondrocytes obtained from OA patients, mesenchymal stromal cells (MSCs) came into the focus for cartilage regeneration due to their immunmodulatory features and regenerative capacities. Even though the benefits of applying MSCs for cartilage repair dominate, also hurdles occur such as rejection of the cells after transplantation into the defect, teratoma formation or low differentiation capability of MSCs into the desired tissue (Toh et al., 2017). Therefore, development of cell free approaches mediating beneficial properties of MSCs are coming into the focus of regenerative medicine research. Extracellular vesicles (EVs) are nanosized particles which transport bioactive molecules (e.g., proteins, lipids, messenger RNA (mRNA), micro RNA (miRNA)) from one cell to another representing a possibility how cells can communicate with each other. EVs are present in all body fluids and are also found in cell conditioned media. In addition, (pathological) alterations of the cellular environment are reflected on the cargos transported within EVs making them excellent biomarkers of various diseases. Several preclinical studies have already demonstrated that EVs play a major role in mediating regenerative effects of MSCs rather than the cells themselves. In this review we give an overview about the latest results of *in vitro* and *in vivo* experiments involving EVs from MSCs in order to improve cartilage regeneration and prevention of degradation. Attention will also be turned to the application of MSC derived EVs in the context of inflammatory arthritis and their application as potential biomarkers for OA especially by harboring miRNAs.

## Current Treatment Options of Osteoarthritis

The degenerative disease Osteoarthritis (OA) affects more than 15% of the population worldwide resulting in strong limitations of performing daily tasks. Besides obesity, trauma and sports injuries, age is the main risk factors of OA. “It affects about 10% of men and 18% of women over the age of 60 (Pourakbari et al., 2019)”. “OA is hallmarked by progressive articular cartilage loss, low-grade synovitis and alterations in the subchondral bone and periarticular tissues (Tofiño-Vian et al., 2018a)”. Currently, treatment options focus on the alleviation of the symptoms such as swelling or pain but therapies to arrest the progression of OA are not available. Clinical treatment options for OA are associated with numerous drawbacks. Reparative techniques including microfracture, in which small holes are drilled into the subchondral bone evoking bleeding and therefore transportation of MSCs from the bone marrow to the cartilage defect, often lead to formation of fibrocartilage which possesses inferior mechanical properties as articular cartilage. The application of osteochondral drafts is often limited by the availability of donor tissue and is connected to a high donor morbidity (Jakob et al., 2002). Another focus lies in the application of cells to treat cartilage defects. Autologous chondrocytes are used during autologous chondrocyte implantation (ACI) in which patient’s cartilage that is not affected by OA is harvested, chondrocytes are isolated and expanded *ex vivo* and are re-implantated into the defected area of the cartilage. Drawbacks of this technique are that it takes a long time, patients have to undergo at least two surgeries, chondrocytes have a limited shelf-life and it is associated with graft delamination and insufficient cartilage regeneration (Harris et al., 2011). “Besides surgical procedures, non-operative treatments including the administration analgesics and non-steroidal anti-inflammatory drugs (NSAIDs) for pain reduction are applied (Gore et al., 2011)”. If patients do not respond to these pharmacological treatments, “intra-articular injections of corticosteroids or hyaluronic acid can be” applied. Although promising results for a short period of time (1-6 months) regarding pain relief as well as amelioration of functionality have been reported, corticosteroids can support further joint degradation. Furthermore, these agents have to be applied at least every 6 months for multiple times (Richards et al., 2016). In advanced stages of OA, in which the above treatments cannot be applied anymore, total joint replacement is the last resort but this procedure is also associated with increased risk of surgical complication, high donor morbidity and limited implant lifetime of around 20 years (Ahmed and Hincke, 2010). Due to all these limitations of cartilage repair techniques, there is an urgent need to develop new strategies which not only relieve patients from pain and inflammation but also lead to restoration of a mechanical functional cartilage tissue.

## Mesenchymal Stromal Cells for the Treatment of Osteoarthritis

The application of MSCs to treat cartilage defects has become the gold standard for more than a decade. A variety of cellular sources have already been reported from which MSCs can be obtained “including bone marrow, adipose tissue, skin and dental pulp” but also perinatal tissues including the amnion, amniotic fluid, umbilical cord and Wharton’s jelly (Heldring et al., 2015). Independent from their source and isolation method, MSCs have to fulfill certain minimal criteria in order to be considered as MSCs which were defined by the International Society for Cellular Therapy in 2006 (Dominici et al., 2006). These criteria include plastic adherence capacity, trilineage multipotency (adipocyte, osteoblast and chondrocyte) as well as “expression of CD73, CD90 and CD105 and the lack of the expression of hematopoietic cell surface markers CD45, CD34, CD14, CD11b, CD79α, CD19 and HLA-DR.”

Due to their multilineage potential and low immunogenicity, MSCs became attractive candidates for the repair of musculoskeletal disorders. Beneficial effects of MSCs have been verified in animal models and clinical studies of rheumatoid arthritis and OA (Wang et al., 2013; Vega et al., 2015; Freitag et al., 2016; Franceschetti and De Bari, 2017), but also in bone (Iaquinta et al., 2019; Marolt Presen et al., 2019), tendon (Jo et al., 2020) and skeletal muscle regeneration (Klimczak et al., 2018). Nevertheless, many studies also revealed that the engraftment of MSCs and subsequent differentiation into the desired cell types were seldomly achieved (Wyles et al., 2015). Another study showed that after application to the target tissue MSCs rapidly dissapeared but they were still able to deliver “chondroprotective and immunomodulatory effects” (ter Huurne et al., 2012). Therefore, the mode of action of many MSC-based therapies could be explained by the secretion of paracrine factors as only a small percentage of MSCs remained at the site of injury. Once inside this injury, MSCs respond to environmental signals such as pro-inflammatory cytokines by secreting factors including cytokines and chemokines, to establish a regenerative environment. By reducing the proliferation of immune cells, MSCs further influence the immune response locally (Aggarwal and Pittenger, 2005). Proteomic analysis identified proteins present in the conditioned medium of MSCs, many of them explaining their beneficial effects for cartilage repair: anti-inflammatory cytokines and chemokines leading to “downregulation of inflammatory cytokines including interleukin (IL)-1β, IL-6 and IL-8” secreted by OA cells as well as protease inhibitors reducing the expression of matrix metalloproteinase (MMP)-1 and MMP-13 (Richards et al., 2016; Ruiz et al., 2016). “Moreover, growth factors known to be involved in cartilage repair and chondrogenesis such as transforming growth factor (TGF)-β, insulin growth factor (IGF)-1, basic fibroblast growth factor (bFGF), vascular endothelial growth factor (VEGF), and epithelial growth factor (EGF) have been identified to be present in the MSCs’ secretomes (Murphy et al., 2013; Ruiz et al., 2016)”. Therefore the MSC conditioned medium can be applied to circumvent drawbacks connected with the therapeutic application of MSC including donor variations, “extensive *ex vivo* expansion of MSC prior to transplantation, induction of senescence, loss of proliferation potential and reduced differentiation capacity beyond 10–20 population doublings (Siddappa et al., 2007).”

## Mesenchymal Stromal Cell-Derived Extracellular Vesicles for the Treatment of Osteoarthritis

Extracellular vesicles (EVs) are released by cells and can be seen as communication media with which physiological and pathophysiological signals are exchanged between various cell types (Lener et al., 2015). They are released by almost all cell types including immune cells, connective tissue cells (epithelials cells, fibroblasts), endothelial cells, neuronal cells, stromal cells but also pathological cells such as tumor cells (Rani et al., 2015). Due to this heterogeneity of cells of origin, EVs can “be found in almost all kinds of body fluids including blood, saliva, urine, milk, amniotic fluid” and synovial fluid which make them also excellent biomarkers for various diseases (Rani et al., 2015; Hock et al., 2017; Katsiougiannis et al., 2017). In a review by Cai et al. (2020) EVs derived from different MSC sources (bone marrow, umbilical cord, adipose tissue) and embryonic mesenchymal stromal cells were compared especially regarding their therapeutic application. It was demonstrated that EVs from specific sources are more appropriate for specific clinical diseases summarized in this review. For example, to treat OA, the tissue of choice to derive MSC-EVs is mostly bone marrow. EVs from all four tissues were able to promote angiogenesis and EVs from bone marrow MSCs, human umbilical cord MSCs and human embryonic MSCs were able to induce tissue repair. Furthermore, proteomic analysis showed that EVs from different MSC sources shared nearly half of all proteins (van Balkom et al., 2019). Moreover, comparing EVs secreted from MSCs derived from pluripotent stem cells (PD-MSCs) with EVs from their parental induced pluripotent stem cells (iPSC) revealed, that iPSC EVs transport proteins which regulate RNA and miRNA stability and protein sorting, whereas PD-MSC EVs “are rich in proteins that organize extracellular matrix, regulate locomotion, and influence cell-substrate” (La Greca et al., 2018). It can be concluded that while PD-MSCs differentiate, their EVs are enriched with a more specific set of proteins compared with EVs from their parental iPSCs.

**FIGURE 1:**
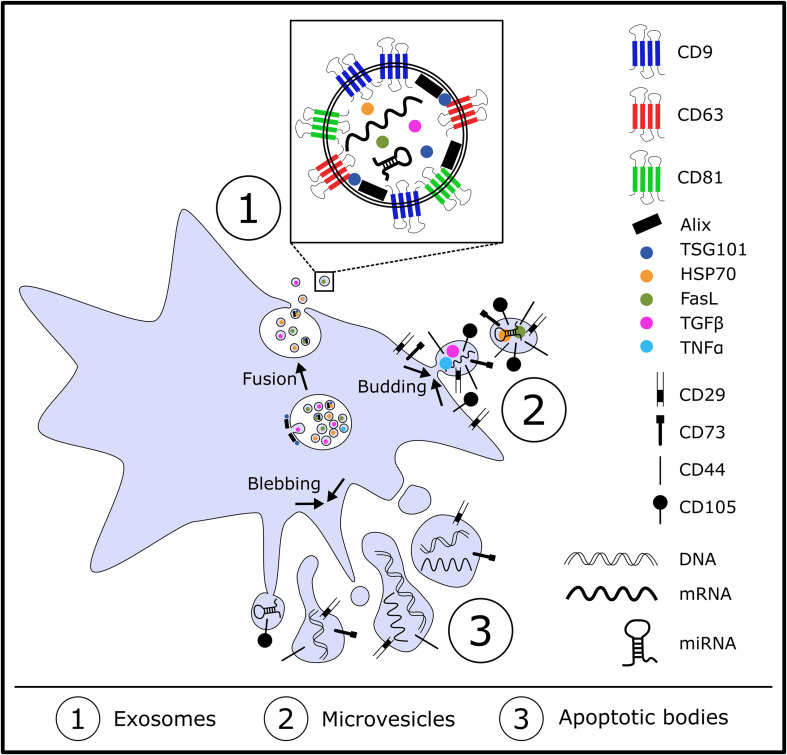
Overview of the biogenesis of exosomes, microvesicles and apoptotic bodies.

“Based on their biogenesis and size, EVs are classified into exosomes, microvesicles and apoptotic bodies (Kalra et al., 2012)” (Figure 1). They are all enclosed by a lipid bilayer, ranging from 30 to 5000 nm in diameter depending on their biogenesis pathway (Esa et al., 2019). Exosomes are ranging between 30 to 120 nm and are generated as intraluminal vesicles in endosomal compartments called multivesicular bodies (MVB) which are a result from endosomal membrane invagination. Within these MVB, intraluminal vesicles (ILV) are present which contain specific “nucleic acids, proteins and lipids”. After fusion of the MVB with the plasma membrane, exosomes are secreted upon exocytosis into the extracellular space. Exosomes are characterized by proteins of the endosomal origin which play a role in the biogenesis of exosomes including Alix and Tsg101. These two proteins are part “of the endosomal sorting complex required for transport (ESCRT), a machinery involved in membrane remodeling and scission in many processes, including cytokinesis”, leading to the formation of ILVs (Hurley, 2015). Although there is still no consensus concerning specific protein markers for different EV types, exosomes tend to be enriched with tetraspanins (CD9, CD63, CD81), which are localized to internal membranes (Escola et al., 1998) as well as heat shock proteins (HSPs) including HSP70 and small GTPases (Urbanelli et al., 2019). Microvesicles are reported to have a size between 100 to 1000 nm and “are generated by outward budding and fission of the plasma membrane.” Therefore “microvesicles harbor cell surface proteins such as receptors, integrins and tetraspanins at relatively low density as well as P-selectin, metalloproteinase MT1-MMP, the two glycoprotein receptors (GP1b and GPIIb/GPIIa) and the integrin Mac-1” l (Meldolesi, 2018). Just like exosomes, microvesicles also carry bioactive molecules including nucleic acids and lipids (Boilard, 2018). However, the knowledge on cellular and molecular mechanisms and events during biogenesis of different EV types with respect to protein sorting is still limited due to technical hurdles during EV isolation and subsequent detailed characterization (Mathieu et al., 2019). With a size between 50 to 5000 nm, apoptotic bodies are a heterogenous population generated during late stages of cell apoptosis (De Jong et al., 2014). “They are generated by membrane blebbing and membrane protrusion and are involved in the clearance of apoptotic material and the modulation of the immune response” (Caruso and Poon, 2018). Exosomes, microvesicles and apoptotic bodies overlap in their sizes but differ in density and especially cargo (Crescitelli et al., 2013). As there are no suitable techniques available to obtain pure EV subtypes and improved isolation and characterization methods are still under investigation, the International Society of Extracellular Vesicles (ISEV) recommends to use the general term extracellular vesicles and to characterize them due to specific physical features “such as size (“small EVs” and “medium/large EVs”), with ranges defined, for instance, respectively, < 100 nm or < 200 nm [small], or > 200 nm [large and/or medium]) or density (low, middle, high, with each range defined), biochemical composition (CD63+/CD81+-EVs, Annexin A5-stained EVs) or descriptions of conditions or cellular origin (podocyte EVs, hypoxic EVs, large oncosomes, apoptotic bodies)” (Thery et al., 2018).

### Isolation and Characterization of Extracellular Vesicles

Based on physical features, various methods can be applied to isolate EVs from biofluids or cell culture supernatants. By using differential ultracentrifugation, ultrafiltration and size exclusion chromatography, EVs are isolated based on their size whereas density gradient ultracentrifugation separates EV subtypes regarding their different densities. Other methods including precipitation or immunoaffinity can also be applied but are connected to limitations such as low purity and protein contaminations in case of precipitation and low yield and high costs regarding immunoaffinity (Table 1). The term yield/recovery defines the total amount of EVs obtained after isolation and several isolation methods are based to recover “the highest amount of extracellular material, despite its vesicular or non-vesicular nature, i.e., whole or near-whole concentrated secretome”, meaning that also free proteins, ribonucleoproteins and lipoproteins can be present in the sample after the isolation process (Thery et al., 2018). On the other hand, isolation methods resulting in high purity/specificity result in EV isolates with as few non-vesicular components as possible. Other commonly used techniques are also depicted with various drawbacks. For example, differential ultracentrifugation is commonly used to isolate EVs even though it is connected to EV aggregation or possible loss of EV functions due to damaged membranes caused by high centrifugal forces (100.000 x g) for several hours. By applying other isolation methods such as size exclusion chromatography, the limitations of differential ultracentrifugation could be avoided. Depending on the volume of the starting material, the size of the SEC columns and the volume of collected fractions, further concentration steps might be conducted as pooling of eluted EV sample fractions may result in low sample concentration which can be critical for further downstream analysis. Additionally, when working with plasma or serum, depletion of lipoproteins and serum proteins like albumin and immunoglobulins is mandatory to avoid biased downstream analysis. Furthermore, when serum is applied as a cell culture supplement it has to be free of EVs before added to the media (Shelke et al., 2014). During the past 20 years, great effort has been put to generate and improve technologies to detect EVs (Coumans et al., 2017). “Quantification of EVs can be conducted by nanoparticle tracking analysis, tuneable resistive pulse sensing or dynamic light scattering, EV morphology can be analyzed by transmission electron microscopy, scanning electron microscopy, cryo-electron microscopy or atomic force microscopy” (Vogel et al., 2016; Sluijter et al., 2018; Wang et al., 2020). “To determine EV specific marker expression, western blotting or flow cytometry with fluorescent counting beads are normally performed (Gyorgy et al., 2012)”.

**TABLE 1:** Comparison of advantages and disadvantages of various methods for EV enrichment.

**Method**	**EV recovery**	**EV specificity**	**EV integrity and functionality**	**Time investment**	**Scalability**	**Costs**
Differential Ultracentrifugation	-) High: Lengthy/very high speed ultracentrifugation without previous, lower-speed steps -) Intermediate: Applying intermediate time/speed with/without washing steps	-) Low: Lengthy/very high speed ultracentrifugation without previous, lower-speed steps -) Intermediate: Applying intermediate time/speed with/without washing steps	High forces could damage EVs	High (up to several hours)	High (for big volumes)	Costly equipment
Ultrafiltration	-) High: low molecular weight cutoff centrifugal filters with no further separation steps -) Intermediate: High molecular cutoff filters	-) Low: low molecular weight cutoff centrifugal filters with no further separation steps -) Intermediate: High molecular cutoff filters	Deformation of vesicles	Low	Low (for small volumes)	Low costs
Size exclusion chromatography	Intermediate	Intermediate	Integrity and biological activity preserved	Low	Intermediate, consecutive concentration steps required due to sample dilution	Low costs
Density gradient ultracentrifugation	Low	High	Preservation of size and shape	High (up to several hours)	Low	Costly equipment
Precipitation Kits	High	Low	Biological activity and integrity retained	Low	Low	Low costs
Immunoaffinity	Low	High	Elution buffers can decrease biological activity	Low	Low	High costs

*Adapted from Thery et al. (2006, 2018) and Pourakbari et al. (2019).*

### Mesenchymal Stromal Cell-Derived Extracellular Vesicles for Cartilage Regeneration

Various uptake mechanisms of EVs are already described but until now it is not totally clear which prerequisites are needed for EVs to be internalized by cells of the joint. One possibility is the presence of EV surface molecules like CD44 which can interact with the hyaluranan matrix of cells such as synoviocytes (Ragni et al., 2019). “Hyaluronan can also be found on the surface of EVs which enables them to interact with proteins and proteoglycans of the extracellular matrix to maintain tissue homeostasis and to contribute to extracellular matrix remodeling and tissue healing (Jiang et al., 2007; Arasu et al., 2017)”.

The positive effects of EVs from several MSC sources on cartilage, subchondral bone, and synovial tissue have already been investigated *in vitro*. The most often used MSC sources for EVs were bone marrow derived MSCs, adipose derived MSCs, embryonic stromal cell derived MSCs, synovial fluid derived MSCs, intra patellar fat pad derived MSCs and induced pluripotent stromal cells and it is assumed, that MSC-derived EVs harbor the same anti-inflammatory and trophic features as their cell of origin. Vonk et al. (2018) demonstrated that OA chondrocytes internalize BMMSC-EVs resulting in an upregulation of aggrecan and type II collagen. At the same time, gene expression of the pro- inflammatory cytokines IL-1, IL-6, IL-8 and IL-17 and cyclooxygenase-2 (COX2) as well as collagenase activity induced by TNF-α were significantly downregulated upon EV uptake. These beneficial effects of BMMSC-EVs were mediated by blocking the phosphorylation of of NF-κβ inhibitor α (IκBα) and subsequently NFκβ activation.

*In vivo* models, in which EVs predominantly derived from MSCs isolated from adult tissues including bone marrow, adipose tissue, synovial membrane or pluripotent cells (embryonic stromal cells) were injected into experimental models of OA, demonstrated that EVs are major players in the recovery of joint injuries and OA. Intra-articular injection of exosomes isolated from embryonic mesenchymal stromal cells (ESC-MSC) into the knee joints of C57BL/6 mice in which the medial meniscus was destabilized led to enhanced synthesis of Collagen II whereas expression of the matrix degrading enzyme ADAMTS5 was reduced compared to PBS treated mice 8 weeks post-surgery (Wang et al., 2017). The same results were obtained when ESC-MSC were injected into the defected area highlighting that exosomes have the same biological activity as their parental cells. In another study, articular cartilage injury was created on the medial femoral condyles of New Zealand White rabbits (Xiang et al., 2018). Administration of BM-MSCs or MVs isolated from these cells resulted in a significant improvement of the 27-point modified O’Driscoll scoring system compared to control groups in which phosphate buffered saline (PBS) was injected into the chondral defect. “This scoring system includes parameters such as percentage of hyaline or articular appearing repair, structural characteristics, freedom from cellular changes of degeneration, freedom from degenerative changes in adjacent cartilage, reconstitution of subchondral bone, and repair of tidemark.” Additionally, BM-MSCs and their respective MVs enhanced collagen deposition and reduced MMP2 expression *in vivo*. “BM-MSCs MVs-mediated cartilage regeneration was mediated by sphingosine-1-phosphate (S1P), a signaling sphingolipid mediating cell proliferation, migration, and barrier function.” S1P was significantly higher in MVs compared to MSCs and presence of S1P neutralizing antibody blocked cartilage defect repair in animals treated with MVs. In another study, Zhang et al. (2016a) established “osteochondral defects on the trochlear grooves of both distal femurs in 12 rats.” These defects were either treated with 100 μg exosomes which were isolated from human embryonic MSCs or PBS. As control, three unoperated animals were used. After 12 weeks post-surgery, animals treated with exosomes showed ameliorated histological scores as well as restoration of the cartilage and subchondral bone similar to unoperated animals.

### miRNAs Transported Within Mesenchymal Stromal Stem Cell-Derived Extracellular Vesicles Can Stimulate Cartilage Regeneration *in vitro* and *in vivo*

Besides proteins and lipids transported within EVs, miRNA have been reported to be mediators in cartilage tissue regeneration and prevention of OA. One of these candidates is miR-140-5p which is known to promote chondrogenic differentiation of MSCs by targeting RalA, an inhibitor of SOX9, leading to its inhibition and therefore activation of SOX9 expression and subsequently ECM secretion (Karlsen et al., 2014; Barter et al., 2015). Exosomes derived from synovial MSCs overexpressing miR-140-5p (SMSC-140s) enhanced the proliferation of articular chondrocytes *in vitro* (Toh et al., 2017). Furthermore, exosomes derived from synovial MSCs which rarely express miR-140-5p (OA + SMSC-Exos) and synovial MSCs overexpressing miR-140-5p (OA + SMSC-140-Exos) were administered into a rat OA model in which the medial collateral ligament and the medical meniscus were completely transected. In the OA group, in which no exosomes were injected into the injury, as well as in the OA + SMSC-Exos group, joint wear and cartilage matrix loss occurred but in the OA + SMSC-Exos group to a lesser extent. In addition, Collagen II was higher expressed compared to the OA group but chondrocytes were arranged in clusters and not in rows as observed in healthy cartilage. Low expression of aggrecan but high type I collagen deposition were detected in the OA + SMSC-Exos group. Overexpression of miR-140-5p in exosomes still resulted in joint wear but in a very mild form compared to the other treatment groups. Also an improvement of the cartilage matrix consisting of type II collagen was observed together with no type I collagen expression. Moreover aggrecan expression was not decreased compared to animals in which no surgery was performed suggesting that OA-SMSC-140-Exos slowed the progression of early OA and prevented severe damage to knee articular cartilage caused by instability of the knee joint. Another miRNA regulating chondrogenesis and cartilage degeneration is miR-92a-3p by targeting WNT5A, a key player in the pathogenesis of OA (Mao et al., 2018b). Additionally, it has also been reported that miR-92a-3p is a major regulator of chondrogenesis and cartilage degradation by directly binding to noggin3, HDAC2, ADAMTS4, and ADAMTS5 (Ning et al., 2013; Mao et al., 2018b). Overexpression of miR-92a-3p in EVs isolated from MSCs promoted proliferation and motility of chondrocytes *in vitro*. Next, MSCs were transfected with either miR-92a-3p or anti-miR-92a-3p and the effects of the corresponding EVs on the chondrogenic differentiation potential of MSCs as well as on articular cartilage was assessed. MSC-miR-92a-3p-EVs induced mRNA and protein expression levels of aggrecan, COL2A1 and SOX9 and decreased the expression levels of COL10A1, RUNX2, MMP13 and WNT5A. Interestingly, MSC-anti-miR-92a-3p-EVs had the exact opposite effect accelerating cartilage matrix degradation and upregulation of WNT5A expression suggesting that MSC-miR-92a-3p-EVs diminish the progression of OA and therefore maintain cartilage stability. These results were further investigated in a collagenase-induced OA model *in vivo*. Administration of MSC-miR-92a-3p-EVs into the injury led to an improvement of col2a1 and aggrecan protein expression compared to the OA group or the group in which MSC-EVs were injected. These effects were also demonstrated on mRNA level by upregulation of COL2A1 and Aggrecan but at the same time WNT5A and MMP13 levels were downregulated demonstrating that MSC-miR-92a-3p EVs lower the progression of early OA and prevent early OA onset *in vivo*. Another candidate for protecting articular cartilage from being degraded is miR-100-5p (Wu et al., 2019). The role of this miRNA, which was present in exosomes derived from infrapatellar fat pad MSCs was investigated *in vitro* and *in vivo*. MSCIPFP-Exos reduced cell apoptosis and expression of catabolic factors including the two main matrix degrading enzymes ADAMTS5 and MMP13, but stimulated matrix synthesis demonstrated by upregulation of Collagen 2 in chondrocytes *in vitro*. Exosomal RNA-seq revealed, that miR-100-5p was one of the strongest miRNAs expressed in exosomes. One target of miR-100-5p is mTOR which negatively regulates autophagy. Binding of miR-100-5p to the 3′untranslated region of mTOR resulted in downregulation of mTOR mRNA also proven by reduced phosphorylation of its target p70S6K. At the same time, expression of autophagy-related protein LC3 was induced. *In vivo*, intra-articular injection of MSCIPFP-Exos into C57BL/6 mice in which OA was induced by surgical destabilization of the medical meniscus resulted in improvement of the severity of OA demonstrated by gait analysis. Interestingly, when antagomir-miR-100-5p was injected into the joint, effects observed with MSCIPFP-Exos were reversed indicating that miR-100-5p plays a major role in the beneficial outcome of MSCIPFP-Exos *in vivo*.

### The Role of Mesenchymal Stromal Cell-Derived Extracellular Vesicles in Inflammatory Arthritis

Inflammation is one of the biggest trigger of OA pathogenesis and progression. Pro-inflammatory cytokines including IL-1β and TNF-α, which are mainly secreted by pro-inflammatory M1 macrophages, induce a variety of inflammatory mediators such as cytokines, chemokines, nitric oxide (NO), prostaglandin E2 (PGE2) and degradative enzymes and high levels of these cytokines can be detected in the synovial fluid of OA patients (Chevalier, 1997; Goldring and Otero, 2011). They are promoting the catabolic processes of OA by inducing the expression of matrix degrading enzymes shifting the equilibrium of homeostasis toward catabolism, leading to the degradation of cartilage.

#### Mesenchymal Stromal Cell-Derived Extracellular Vesicles Have Chondroprotective Properties in an OA Simulated Inflammatory Environment

To simulate and inflammatory environment as present in OA *in vitro*, MSCs are pre-treated with the inflammatory cytokine IL-1β prior to EV isolation. In a study by Kato et al. (2014), human synovial fibroblast (SFB) were stimulated with IL-1β, exosomes were isolated and articular chondrocytes were treated with these vesicles. As control, articular chondrocytes were cultured with exosomes from untreated SFB. When SFB were pre-treated with IL-1β, more exosomes were released compared to untreated SFBs. Exosomes isolated from SFB which were pre-treated with IL-1β stimulated OA-like changes reflected in gene expression of human articular chondrocytes as well as cartilage degradation demonstrated by elevated expression levels of MMP-3, IL-6 and VEGF compared to control groups. Furthermore, these exosomes enhanced angiogenic activity including migration and tube formation in HUVEC. Enhanced angiogenesis is observed in most tissues in OA joints. In another study, chondrocytes from OA patients were treated with IL-1β and co-cultured with EVs isolated from adipose derived MSCs (ASC) (Tofiño-Vian et al., 2018b). ASC-MVs and exosomes decreased the levels of TNF-α, IL-6, NO and PGE2, the latter due to a downregulation of COX2 and microsomal prostaglandin E synthase-1. The same effect was observed on OA explants. The group demonstrated in another study that ASC-EVs possess beneficial effects on OA osteoblasts (Tofiño-Vian et al., 2017). They stimulated cells with IL-1β and exosomes as well as MVs from ASCs were able to reduce senescence- associated β-galactosidase activity and the accumulation of γH2AX foci. As for OA chondrocytes, exosomes and MVs were able to downregulate IL-6 and PGE2 levels in OA osteoblasts. The involvement of other pathways rather than the NFκβ-COX2 pathway in an inflammation induced setting was demonstrated by Qi et al. “In their study they investigated the significance of BMSCs derived exosomes (BMSC-Exos) on the viability of chondrocytes under normal and IL-1β induced inflammatory conditions (Qi et al., 2019)”. IL-1β induced apoptosis, decreased viability and changed the mitochondrial membrane potential of chondrocytes but when BMSC-Exos were added, these effects could be reverted. They demonstrated that PI3K/AKT and ERK1/2 pathways played a crucial role, as under inflammatory conditions p38 and ERK1/2 were phosphorylated whereas Akt was inhibited. “ERK1/2 is one of the key signal pathways associated with responses to mitogenic activation (Chang and Karin, 2001) and p38 contributes to IL-1β induced apoptosis of chondrocytes (Phornphutkul et al., 2008)”. On the other hand, the PI3K/AKT pathway is one of the master regulators of cellular growth, protein synthesis and cell survival (Fresno Vara et al., 2004; Martini et al., 2014). Interestingly, in the presence of BMCS-Exos in combination with IL-1β, phosphorylation of p38 and ERK was reduced whereas the phosphorylation of Akt was stimulated compared to the IL-1β only treated group.

Zhang et al. (2019) established a model of temporomandibular joint OA (TMJ-OA)*in vivo* via MIA injection resulting in “sustained inflammation with increased expression of pro-inflammatory cytokines” leading to pain and degeneration of the joint. Three groups of animals were created: OA + PBS, OA + Exosomes isolated from “immortalized E1-MYC 16.3 human embryonic stem cell-derived MSCs”, and a Sham group. Two weeks after induction of OA, exosomes were administrated weekly to the respective group for 2, 4 or 8 weeks. Exosomes were able to regenerate early TMJ-OA mainly through decreasing inflammation leading subsequently to reduced pain levels and tissue degeneration. On the other hand, exosomes were able to induce proliferation as well as matrix synthesis in order to re-establish TMJ osteochondral tissues. This restoration was especially achieved at the end of week 8 as exosomes were able to rebuilt condylar structures similar as in animals which did not receive surgery.

#### Mesenchymal Stromal Cell-Derived Extracellular Vesicles Influence Cells of the Immune System to Restore Cartilage Integrity

Synovial inflammation is recognized as a hallmark of OA with deleterious impact on joint function. “Synovial inflammation leads to synovial lining hyperplasia, fibrosis, neo-vascularization and the appearance of macrophages (Sellam and Berenbaum, 2010).” By releasing pro-inflammatory mediators, monocytes and macrophages are the main triggers of inflammation promoting progression of OA. “Classical M1 and alternative M2 activated macrophages represent two extremes of a dynamic state of activation. Classical activated M1 macrophages, induced by interferon-γ alone or in combination with microbial stimuli such as lipopolysaccharide (LPS) and/or inflammatory cytokines, exert pro inflammatory activities by secreting cytokines such as TNF-α, IL-12, and IL-1β and inhibit chondrogenesis of MSCs via IL-6 (Drexler et al., 2008; Fahy et al., 2014; Martinez and Gordon, 2014; Lo Sicco et al., 2017)”. On the other hand, cytokines including IL-4 and IL-13 induce and alternative activation of M2 macrophages, triggering resolution of inflammation. Several studies indicated that EVs from various cell sources are able to induce the polarization from M1 to M2 in inflammatory arthritis *in vitro* and *in vivo*. Microparticles and exosomes isolated from murine bone marrow MSCs were able to inhibit macrophage activation indicated by reduced levels of F4/80^+^ macrophages expressing CD86, MHCII or CD40 markers. In addition, reduced activation of macrophages was demonstrated by lower levels of TNF-α and elevated levels of IL-10 upon treatment with microparticles and exosomes (Cosenza et al., 2017). Similar effects were also shown when bone marrow-derived macrophages where treated with EVs isolated from adipose derived MSCs under normoxic and hypoxic conditions (Lo Sicco et al., 2017). “In standard conditions, macrophages expressed significant higher levels of the pro-inflammatory M1-like markers Ly6C, CD11b, CD40 and CD86 compared to EV treated cells and did not express any of the M2 markers like scavenger receptor CD36, the mannose receptor CD206 or the α_*v*_β_3_ integrin CD51.” Treatment with either EV^*Normo*^ or EV^*Hypo*^ for 72 hours resulted in a change of recipient macrophages toward an anti-inflammatory phenotype. Interestingly, EVs which were released under hypoxic conditions downregulated the expression of CD86 and the activation marker CD11b to a stronger extend compared to EVs released under normoxia. Moreover, it was shown that EVs isolated from human adipose derived MSCs suppressed the expression of COX-2, IL-1β, IL-6, and TNF-α in RAW264.7 macrophages as well as stimulated their polarization from M1 to M2 (Woo et al., 2020). The ability of EVs to modulate immune reactivity in cartilage defects was also investigated *in vivo*. “In a study by Zhang et al. (2018a), osteochondral defects were created on the trochlear grooves of the distal femurs of rats with a drill bit followed by intra-articular injection of exosomes isolated from human embryonic stromal cell derived MSCs.” As vehicle control, PBS was administrated. With regards to M2 macrophages, a higher number of CD163^+^ cells were found in the cartilage and the overlying synovium of exosome-treated defects compared to control groups. On the contrary, fewer CD86^+^ cells representing M1 macrophages were detected in both cartilage and synovium of exosome-treated groups in comparison to the vehicle groups. This effect was observed for 12 weeks. In addition to the histological examination of the cartilage defects, synovial fluid samples of the respective animals were collected. In exosome-treated animals, reduced levels of the M1 associated cytokines IL-1β and TNF-α were detected compared to control animals at week 6 but this was not the case for IL-6. Furthermore, Woo et al. (2020) evaluated the role of EVs from human adipose derived MSCs in a monosodium iodocetate (MIA) induced rat model of OA. MIA administration into the knee joint induced cartilage degradation and also affected the perimeniscal synovium and the infrapatellar fat pad. “Injection of EVs prevented fibrotic deposition and blood vessel formation and was also able to restore the adipocyte-rich appearance of the infrapatellar fat pad. In addition, EVs inhibited the infiltration of M1 macrophages at the OA synovium and reduced the expression of IL-1β in both the synovium and the cartilage in the MIA-induced OA model.” The fact that EVs not only have an impact on macrophages but also on other immune cells was demonstrated in a study by Cosenza et al. (2018). Microparticles and exosomes were isolated from murine bone marrow derived MSCs and their effects on T and B lymphocytes were studied *in vitro*. Microparticles as well as exosomes decreased the proliferation of T lymphocytes indirectly through Tr1 and Treg induction. Furthermore, plasmablast differentiation was reduced in the presence of microparticles and exosomes but also in the presence of MSCs indicated by reduced levels of IgG produced in the coculture supernatant. *In vivo*, exosomes but not microparticles were able to reduce signs of arthritis in a collagen-induced arthritis model including paw swelling and clinical scores up to 35% compared to control mice. Interestingly, microparticles and exosomes lost their immunomodulatory properties after freeze thawing circles at −80°C most probably due to disruption of their membranes.

## EV-Associated miRNAs as Potential Diagnostic Biomarkers for Osteoarthritis?

MicroRNAs (miRNAs) are small single-stranded non-coding RNA molecules with a length of around 22 nucleotides (nt) on average. They are mainly generated from primary miRNAs (pri-miRNAs) that are genetically encoded and transcribed via RNA-polymerase II similar to protein-coding messenger RNAs (mRNAs) (Lee et al., 2004; Carthew and Sontheimer, 2009). Pri-miRNAs are processed by the RNAse Drosha (Lee et al., 2003) into pre-miRNA hairpin intermediates and exported into the cytoplasm (Lund et al., 2004) to be cleaved into around 22 (nt) long RNA duplexes by the RNAse DICER (Knight and Bass, 2001). One of the strands is degraded, while the other represents the mature miRNA and is bound by Argonaute family proteins into a ribonucleoprotein complex called miRNA-induced silencing complex (miRISC) (O’Brien et al., 2018). The mechanism of action of miRNAs in this complex is binding to complementary target sequences in the 3‘ untranslated region of mRNAs. Thereby, the seed region (Bartel, 2009) of a miRNA forms a partial RNA duplex with the mRNA that results in mRNA degradation or halting of translation depending whether the base pairing is a full or partial match, respectively. These effects negatively regulate gene expression post-transcriptionally mainly via inhibition of translation of target mRNAs (Bartel, 2009; Carthew and Sontheimer, 2009). However, there are reports that indicate miRNA-dependent activation of gene expression promoting translation or even transcription (Vasudevan and Steitz, 2007; Vasudevan, 2012).

Besides biosynthesis from transcripts of dedicated miRNA genes, miRNAs can be synthesized from introns of protein-coding genes (miRtrons) or other non-coding RNAs and even tRNAs can be processed into mature miRNAs. Isoforms of canonical miRNA genes (isomiRs) can exist and a single pre-miRNA can give rise to up to 5 or more different functionally relevant mature miRNA sequences (Desvignes et al., 2015). This poses a challenge to establish proper nomenclature, however, harbors a comprehensive functional repertoire to finetune cellular metabolism under physiologic and pathologic conditions, as well as offers a huge treasure trove of potential biomarkers. Gene regulation by miRNAs is highly pleiotropic. A single miRNA might target a variety of target genes involved in different pathways, while a single mRNA 3′-UTR might be targeted by different miRNAs. These interdependent effects emphasize the complexity of the regulatory network, which is set up by the miRNAome (Shirdel et al., 2011). Therefore, attention has to be paid when analyzing and interpreting the effect(s) of single miRNAs which might mediate opposing functional consequences such as pro- and anti-fibrotic functions in the case of miR-192 (Jenkins et al., 2012).

### Deregulated miRNAs in OA Are Potential Biomarkers in Cartilage, Blood, and Synovial Fluid

**FIGURE 2:**
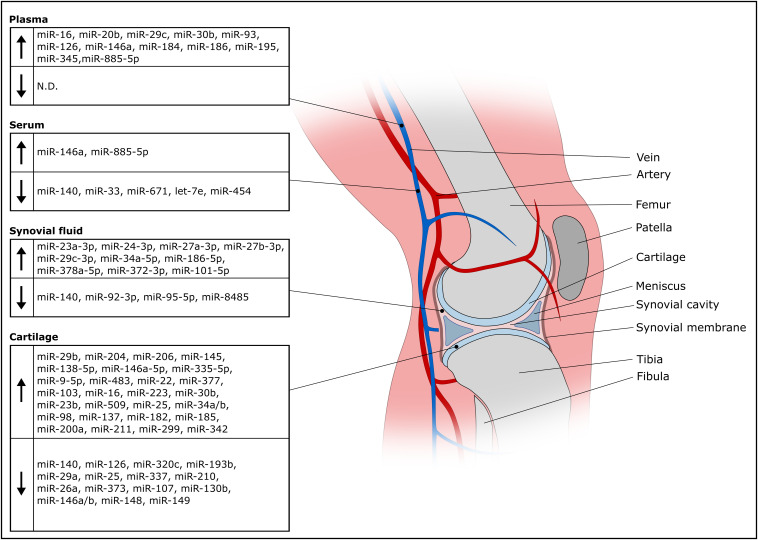
Overview of deregulated miRNAs in OA isolated from olasma, serum, synovial fluid and cartilage.

A miRNA might qualify as biomarker for OA either if it is found in lower concentration in the disease condition or if its concentration increases relative to other miRNAs involved in the regulatory network of chondrogenic gene expression. In addition, specific miRNAs might characterize distinct phases of OA, whether the given miRNA is involved in onset, progression or late stage of disease. Figure 2 gives an overview about deregulated miRNAs in OA isolated from plasma, serum, synovial fluid and cartilage.

OA cartilage is characterized by reduced expression of miR-140 (Iliopoulos et al., 2008; Miyaki et al., 2009), which facilitates onset of aging-related cartilage damage and renders cartilage susceptible to inflammation (Miyaki et al., 2010; Yang et al., 2018). Cartilage-protective effects are mediated by miR-140 via targeting ADAMTS5, MMP13 and HDAC4 driving expression of COL2A1 and ACAN (Tuddenham et al., 2006; Miyaki et al., 2009; Si et al., 2020). OA progression is accompanied by decreased miR-337 expression and loss of miR-337 shifts the equilibrium from anabolism of cartilage tissue to bone formation, (Zhang et al., 2018b). Studies frequently find more upregulated than downregulated miRNAs in OA (Ntoumou et al., 2017; Coutinho, de Almeida et al., 2019). This coincides with the idea that a couple of miRNAs might be sufficient to stabilize a chondrotypic gene regulatory network and many other miRNAs might interfere with the nodes of the network to dysregulate the chondrocyte phenotype. Expectedly, numerous studies report effects of upregulated miRNAs in OA cartilage. A study identified 142 differentially expressed miRNAs in OA cartilage, with miR-206, a muscle-specific miRNA (Luo et al., 2013), having the largest fold change among 91 upregulated miRNAs (Coutinho, de Almeida et al., 2019).

Other studies try to define a set of miRNAs as biomarkers of OA. Four miRNAs (miR-138-5p, miR-146a-5p, miR-335-5p, miR-9-5p) were significantly upregulated in OA cartilage compared to a control group (Kopańska et al., 2017). Another work reports a set of 16 differentially regulated miRNas in a panel of 365 miRNAs including 9 upregulated miRNAs (miR-483, miR-22, miR-377, miR-103, miR-16, miR-223, miR-30b, miR-23b, miR-509) and 7 downregulated miRNAs (miR-29a, miR-140, miR-25, miR-337, miR-210, miR-26a, miR-373) (Iliopoulos et al., 2008). Later, a study identified 17 differentially expressed miRNAs out of 157 tested miRNAs having 12 upregulated (miR-9, miR-25, miR-34a, miR-34b, miR-98, miR-137, miR-182, miR-185, miR-200a, miR-211, miR-299, miR-342) and 5 downregulated miRNAs (miR-107, miR-130b, miR-146, miR-148, miR-149) (Jones et al., 2009). While miR-146 was found among the downregulated miRNAs in the Jones et al. study, which most probably refering to the miR-146a/b family having the same seed sequence and differing by only 2 nucleotides (Rusca and Monticelli, 2011), others found elevated miR-146a/b in OA cartilage (Budd et al., 2017; Skrzypa et al., 2019) or synovial tissue (Li et al., 2011). Considering the involvement of miR-146a/b in anti-inflammatory processes (Rusca and Monticelli, 2011) and the differential expression in OA tissue samples, presence of miR-146a/b might help to differentiate early from later OA stages (Yamasaki et al., 2009).

Reported cartilage miRNA biomarker profiles are often highly variable and non-overlapping. This might result from different sampling strategies, processing and handling, as well as stage of disease. Screening OA cartilage for biomarkers requires a tissue biopsy, which is contra-productive for therapy as a biopsy takes away already dwindled cartilage. Non-invasive diagnostic procedures are available to assess disease stages (Kellgren and Lawrence, 1957), so there is no requirement for miRNA profiling in OA cartilage for diagnosis, although it might provide a lot of information concerning the inflammatory status for example. To circumvent tissue biopsies and prevent complications, miRNA profiling in plasma or serum of OA patients has been investigated. The procedure is minimally invasive and more standardizable. Beside elevated miR-146a in OA cartilage, the same authors report elevated miR-146a in serum among a panel of 18 miRNAs involved in cartilage homeostasis (Skrzypa et al., 2019). A screen for OA miRNA serum biomarkers resulted in identification of miR-140, miR-33 and miR-671 being significantly downregulated in 279 differently expressed miRNAs in a 2549 miRNA panel (Ntoumou et al., 2017). An earlier study screened 377 miRNAs in OA serum samples and identified 12 differentially expressed miRNAs (miR-122, miR-25, miR 28-3p, miR-93, miR-140, miR-191, miR-342-3p, miR-146b, miR-454, miRNA-885-5p, let-7b, let-7e). miRNA-454, miRNA-885-5p and let-7e showed predictive capacity and circulating let-7e inversely correlated with severity of knee or hip osteoarthritis (Beyer et al., 2015).

A plasma miRNA signature of 12 overexpressed miRNAs in OA included miR-16, miR-20b, miR-29c, miR-30b, miR-93, miR-126, miR-146a, miR-184, miR-186, miR-195, miR-345 and miR-885-5p (Borgonio-Cuadra et al., 2014), however, miRNAs downregulated in plasma during OA has not been thoroughly investigated. Serum and plasma exhibit very disparate OA miRNA profiles, only miR-885-5p was found upregulated in serum and plasma miRNAs as described above. Therefore, blood processing before biomarker analysis has to be considered, given the fact that clotting and platelet activation impact circulating miRNA profiles (Italiano et al., 2010; Burnouf et al., 2014). However, although there are reports that suggest differently expressed plasma miRNA in OA versus healthy controls, others report that there is no difference of a 177 miRNA panel between healthy and OA plasma miRNA profiles (Aae et al., 2020). These authors explicitly focused on extracellular vesicle (EV) associated miRNAs. EVs can package miRNAs and protect them from degradation (Valadi et al., 2007). For this reason, EVs might be a valuable source for biomarkers as EV synthesis is considered a tigthly regulated process (Palmulli and van Niel, 2018). Nevertheless, EV-associated miRNAs are only a proportion of extracellular miRNA found in plasma or serum and very few studies so far clarified whether the identified miRNAs are EV-associated in the context of biomarker discovery. Tissue damage causes release of cytosolic material including miRNAs which are protected from degradation as well via association with RNA-binding proteins (Turchinovich and Burwinkel, 2012) or binding to HDL (Vickers et al., 2011). Therefore, one might question whether whole plasma or serum miRNA profiles are truly indicative of OA pathogenesis or represent non-specific debris resulting from (patho-)physiologic activity throughout the body (Turchinovich et al., 2016). By looking at miRNAs associated with enriched or isolated EVs, a diagnostic application of EV-associated miRNAs in OA might be less affected by potential contaminations with other extracellular RNA.

One study compared plasma and synovial fluid miRNAs in different arthropathies (Murata et al., 2010). They found that miRNA profiles were very distinct from plasma miRNA levels and could reflect conditions in the synovial cavity. Synovial fluid (SF) is probably the best option to look for miRNAs as OA biomarkers. It is in direct contact with affected joint tissues which secrete EVs into SF (Kato et al., 2014; Murphy et al., 2018; Ni et al., 2020) and is separated by the synovial membrane from to the circulation, therefore EVs are not diluted away and might be more concentrated than blood-derived biomarkers. Chondrocyte-specific miR-140 is not only decreased in OA chondrocytes, but in SF as well, and expression negatively correlates with OA grade (Si et al., 2016). SF miRNA profiles are able to differentiate early and late stage OA. Seven out of 752 miRNAs (miR-23a-3p, miR-24-3p, miR-27a-3p, miR-27b-3p, miR-29c-3p, miR-34a-5p and miR-186-5p) were increased in late stage compared to early stage OA SF. Two miRNAs (miR-23a-3p, miR-27b-3p) were shown to be released from synovial tissue into SF during inflammation, while three miRNAs (miR-27a-3p, miR-378a-5p, miR-101-5p) were only found in late stage OA (Li et al., 2016).

Precipitated EVs from OA chondrocytes were found to contain high levels of miR-372-3p compared to EVs from normal cells, while cytoplasmic miR-372-3p levels decreased suggesting an EV-associated release (Song et al., 2017a). Similarly, another study found a strong decrease of miR-372 in OA chondrocytes after 6 h stimulation with IL1β, however did not investigate released miRNAs (Akhtar et al., 2010). Increased GSK activity correlated with stronger miR-372-3p release, which is a result of elevated Wnt signaling in OA (Usami et al., 2016). Unfortunately, no study compared miR-372-3p levels in EVs directly isolated from OA synovial fluid versus healthy controls, nevertheless, it could be expected that miR-372-3p is released into the synovial cavity and might be evaluated as biomarker by future studies. In contrast, synovial fluid levels of miR-210 showed a strong positive correlation with early and late stage OA and might serve as biomarker for OA (Xie et al., 2019). Although, these authors investigated total RNA from synovial fluid, others confirmed an association of miR-210 with EVs in the context of OA (Kolhe et al., 2017) and other conditions (Mills et al., 2019). EV-associated miR-95-5p levels released from OA chondrocytes decreased, while overexpressed miR-95-5p promoted chondrogenesis via targeting HDAC2 and HDAC8 (Mao et al., 2018a). Similarly, miR-92-3p was decreased in EVs released from OA chondrocytes targeting HDAC2 as well as WNT5A resulting in decreased ECM synthesis (Mao et al., 2018b). Other miRNAs targeting Wnt signaling include miR-8485. Screening SF for decreased miR-8485, which was released in EVs from healthy chondrocytes in a co-culture system and promoted chondrogenic differentiation, might be involved in cartilage homeostasis and serve as future candidate biomarker (Li et al., 2020). These studies investigated EV-associated miRNAs derived from chondrocytes in culture, however, this might not necessarily reflect an *in vivo* situation. On the other hand, degraded cartilage explants released substantially more EVs than non-degraded explants with differential expression of miR-449a-5p (Ni et al., 2019). Explants are a more physiologic model system than chondrocyte cultures, thus observed differential expression of miRNA profiles will be more representative for biomarker discovery. Nevertheless, the findings need to be confirmed via assessing differential expression of miRNAs in directly isolated SF before claiming diagnostic value of EV-associated miRNAs in OA, similar to a study reporting miR-200c-3p upregulation in OA SF.

Beside the choice of the source material for diagnosis, pre-analytical parameters might influence a miRNA biomarker profile. For example, gender-specific EV-associated miRNA expression was found in SF of OA patients. Female samples showed increased levels of miR-16-2-3p and decreased expression of miR-26a-5p, miR-146a-5p and miR-6821-5p. Male patients had downregulated miR-68678-3p and upregulated miR-210-5p. Only upregulated miR-504-3p was common to both female and male samples (Kolhe et al., 2017). Obesity is not only a risk factor for OA (Lementowski and Zelicof, 2008; Bliddal et al., 2014), but might also impact potential miRNA biomarker profiles. Body mass index (BMI) positively correlated with miR-22 and miR-103 expression in OA cartilage resulting in increased IL-1β and MMP13 expression via PPARA signaling, while miR-25, miR-29a and miR-337 inversely correlated with BMI (Iliopoulos et al., 2008). Nevertheless, miRNA profiles in SF were neither affected by age, gender or BMI (Li et al., 2016). In summary, miRNAs associated with EVs might be used as biomarkers for OA, but future studies are advised to discriminate between sources of biological material, favor biofluids like SF which are in close proximity to diseased tissue and highlight whether identified miRNA profiles are truly EV-associated rather than being derived from other reservoirs of extracellular RNA.

## Extracellular Vesicles for Tissue Engineering Approaches

To keep EVs within the injured cartilage in order to prolong their stimulatory effects, EVs have been mixed with different scaffolds. EVs isolated from iPSC-MSC have been incorporated into a photoinduced imine crosslinking hydrogel tissue patch and after implanting this construct into a rabbit articular defect model, new hyaline cartilage was formed which integrated with the native cartilage (Liu et al., 2017). In another study, EVs isolated from adipose derived MSCs undergoing chondrogenic differentiation were incorporated into a hyaluronic acid hydrogel which was then administered into the joints of rats which were treated with monosodium iodacetate. This approach extenuated proteoglycan degradation (Alcaraz et al., 2019). As the printing techniques also comes into the focus of tissue engineering strategies, a 3D printed cartilage ECM/gelatin methacrylate/EVs construct has been implanted into osteochondral defects of rabbits. This scaffold induced cartilage formation and restored chondrocyte mitochondrial dysfunction (Chen et al., 2019). Moreover, incorporation of human umbilical cord mesenchymal stromal cells derived small EVs into a gelatin methacrylate nanoclay hydrogel promoted cartilage regeneration of full thickness cylindrical cartilage defects created by electrical drill *in vivo* (Hu et al., 2020). Even though only few studies have been published regarding the combination of scaffolds and EVs, it is important not to lose sight of this approach as it may further impact the regenerative capacity of EVs embedded within an environment mimicking components of healthy cartilage.

## Discussion

As the society is aging, the incidence of OA is increasing especially in people over the age of 60 years. As current treatment options for OA are mainly palliative and do not lead to a fully restoration of the function of cartilage, other therapeutic strategies have to be developed. Mesenchymal stromal cells based therapies have been one of the treatment options of choice for the last decades due to their ability to differentiate into the desired tissue as well as due to their immunomodulatory features. Within the last years it has been evidenced that not the cells *per se* but their secretomes play a major role in mediating their regenerative capacity. Besides soluble factors, EVs are the main components found within a cell’s secretome. EVs can be isolated from all body fluids including plasma, urine, amniotic fluid, and synovial fluid as well as from conditioned media. As they carry bioactive molecules such as nucleic acids, proteins and lipids, EVs mediate therapeutic effect in diseased joints by stimulating expression of ECM proteins, enhancing chondrocyte proliferation, inhibiting matrix degrading enzymes and reducing levels of pro-inflammatory mediators.

To maximize therapeutic effects of MSC-derived EVs, exposure of MSCs to biophysical cues, pre-treatment of MSCs with pro-inflammatory stimuli and cell reprogramming for protein or miRNA expression have been explored (Park et al., 2019). For example, MSCs cultured in a 3D scaffold versus conventional 2D culture enhanced EV yield (Zhang et al., 2017), while MSC EVs incorporated in a hydrogel promoted articular cartilage regeneration (Liu et al., 2017). MSC coculture with chondrocytes might be a possibility to obtain a MSC secretome including EVs specifically promoting chondrogenic differentiation (Cooke et al., 2011). Priming MSCs with IL1β enhanced packaging of anti-inflammatory miR-146a into EVs (Song et al., 2017b), which increased therapeutic efficacy and might yield a stronger reduction of inflammation in chondrocytes or the whole joint space (Zhong et al., 2017). Strategies involving more profound engineering approaches like miR-140 overexpression in MSCs to generate miRNA-loaded EVs for inhibiting inflammation in the joint and promoting chondrogenic gene expression (Karlsen et al., 2014, 2016; Huang et al., 2019) might complicate the approval of MSC EVs for clinical use.

Nevertheless, translation of EV research into the clinics is associated with less regulatory hurdles compared to cell-based therapies. EVs can be manufactured in larger quantities and their injection allows an accurate dosing schedule and a better control of treatment (Tofiño-Vian et al., 2018a). Furthermore, drawbacks including immune rejection or transformation of transplanted cells into tumors can be circumvented by using EVs for therapeutic approaches. Like synthetic carriers such as liposomes or artificially generated nanoparticles, EVs possess a lipid bilayer membrane which protects their cargo from degrading enzymes. However, due to their “natural” origin, EVs represent better vehicles for delivering specific agents or molecules compared to synthetic ones. “As EVs have a lower immunogenic potential compared with cells (Lai et al., 2010; Zhang et al., 2014, 2016a,b) applications of allogenic EVs have been reported to be safe and could be administrated in larger quantities.” For these reasons, patients will profit from EV therapies, as a low risk of adverse effects faces a spectrum of favorable outcomes combined with a relative ease of use.

Despite all the promising results in preclinical studies, many questions still remain unanswered: Which impact does the source of MSC have on EVs? Which source is the most appropriate for cartilage regeneration or does the source matter at all? Most studies only focus on one source of MSCs but comparative studies show that for examples EVs isolated from iPSC derived MSCs have a greater therapeutic effect on OA than EVs isolated from synovial membrane-derived MSCs (Zhu et al., 2017). Furthermore, there is a strong need for standardization not only regarding cultivation and characterization of MSCs but more urgently for EVs. Even though the MISEV 2018 guidelines were established in order to give reference points regarding nomenclature, isolation techniques and characterization methods, in the end they are stated as recommendations. As long as there is not an uniform guideline in order to determine “the biogenesis, composition, appropriate delivery technique, *in vivo* stability and distribution, internalization, mechanisms of action, efficacy, long-term actions and most importantly safety of EVs” (Tofiño-Vian et al., 2018a), translation into clinical studies and as a last resort acceptance of EVs as a potential therapy for treating OA will be delayed. There is a need to give millions of people suffering from OA hope that they will not only be relieved from pain but also giving them back their mobility and therefore quality of life and EVs are promising candidates to be these silver linings.

## Author Contributions

ADL and AO wrote the review. SN gave ideas for chapters and revised the review. All authors contributed to the article and approved the submitted version.

## Conflict of Interest

The authors declare that the research was conducted in the absence of any commercial or financial relationships that could be construed as a potential conflict of interest.
